# Benzyl­ethyl­dimethyl­ammonium bromide

**DOI:** 10.1107/S1600536808002481

**Published:** 2008-02-15

**Authors:** Maciej Hodorowicz, Katarzyna Stadnicka

**Affiliations:** a Faculty of Chemistry, Jagiellonian University, Ingardena 3, 30-060 Kraków, Poland

## Abstract

The crystal structure of the title compound, C_11_H_18_N^+^·Br^−^, has been determined as part of an ongoing study of the influence of the alkyl chain length on amphiphilic activity of quaternary ammonium salts. The title salt forms a three-dimensional network of ionic contacts through weak C—H⋯Br hydrogen bonds, with donor–acceptor distances in the range 3.757 (2)–3.959 (2) Å, in which methyl groups serve as donors.

## Related literature

For related literature, see: Ogawa & Kuroda (1997 ▶); Hodorowicz *et al.* (2003 ▶, 2005 ▶); Kwolek *et al.* (2003 ▶); Allen *et al.* (1987 ▶).
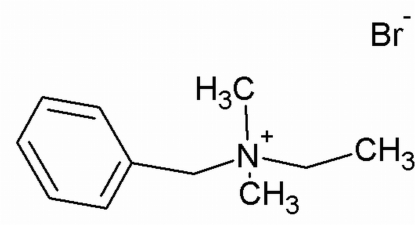


## Experimental

### 

#### Crystal data

C_11_H_18_N^+^·Br^−^
*M* *_r_* = 244.17
Orthorhombic, 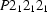

*a* = 6.7765 (1) Å
*b* = 12.5827 (2) Å
*c* = 13.9433 (2) Å
*V* = 1188.90 (3) Å^3^
*Z* = 4
Mo *K*α radiation
μ = 3.42 mm^−1^
*T* = 293 (2) K
0.20 × 0.19 × 0.17 mm

#### Data collection

Nonius KappaCCD diffractometer
Absorption correction: multi-scan (*DENZO* and *SCALEPACK* Otwinowski & Minor, 1997 ▶) *T*_min_ = 0.548, *T*_max_ = 0.594 (expected range = 0.516–0.559)
18366 measured reflections
3874 independent reflections
3483 reflections with *I* > 2σ(*I*)
*R*_int_ = 0.041

#### Refinement

*R*[*F* ^2^ > 2σ(*F* ^2^)] = 0.027
*wR*(*F* ^2^) = 0.065
*S* = 1.07
3874 reflections
119 parameters
H-atom parameters constrained
Δρ_max_ = 0.26 e Å^−3^
Δρ_min_ = −0.49 e Å^−3^
Absolute structure: Flack (1983 ▶), with 1627 Friedel pairs
Flack parameter: 0.002 (9)

### 

Data collection: *COLLECT* (Nonius, 2000 ▶); cell refinement: *SCALEPACK* (Otwinowski & Minor, 1997 ▶); data reduction: *DENZO* (Otwinowski & Minor, 1997 ▶) and *SCALEPACK*; program(s) used to solve structure: *SIR92* (Altomare *et al.*, 1994 ▶); program(s) used to refine structure: *SHELXL97* (Sheldrick, 2008 ▶); molecular graphics: *ORTEP-3 for Windows* (Farrugia, 1997 ▶) and *DIAMOND* (Brandenburg, 2006 ▶); software used to prepare material for publication: *SHELXL97*.

## Supplementary Material

Crystal structure: contains datablocks I, global. DOI: 10.1107/S1600536808002481/cf2179sup1.cif

Structure factors: contains datablocks I. DOI: 10.1107/S1600536808002481/cf2179Isup2.hkl

Additional supplementary materials:  crystallographic information; 3D view; checkCIF report

## Figures and Tables

**Table 1:** Hydrogen-bond geometry (Å, °) *Cg*1 is the centroid of the benzene ring.

*D*—H⋯*A*	*D*—H	H⋯*A*	*D*⋯*A*	*D*—H⋯*A*
C4—H4*A*⋯Br1	0.97	2.81	3.757 (2)	164
C2—H2*A*⋯Br1^i^	0.96	2.96	3.850 (3)	154
C4—H4*B*⋯Br1^i^	0.97	2.96	3.832 (2)	151
C3—H3*B*⋯Br1^i^	0.97	3.08	3.950 (2)	151
C3—H3*A*⋯Br1^ii^	0.97	3.19	3.959 (2)	138
C1—H1*C*⋯Br1^iii^	0.96	2.99	3.766 (2)	139
C2—H2*C*⋯*Cg*1^ii^	0.96	2.69	3.526	145

Symmetry codes: (i) 
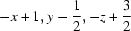
; (ii) 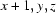; (iii) 
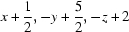
.

**Fig. 1.**
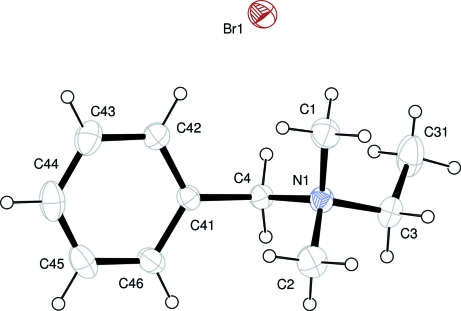
ORTEP-3 (Farrugia, 1997) drawing of the asymmetric unit with atom labels. Displacement ellipsoids of non-H atoms are drawn at the 30% probabilty level.

**Fig. 2.**
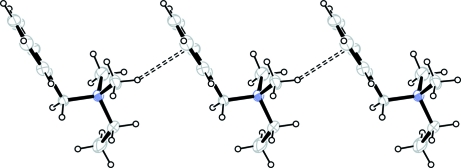
Chain of benzyldimethylethylammonium cations along [100] projected onto (010). The chain is formed due to C—H···π interactions (ORTEP-3; Farrugia, 1997).

**Fig. 3.**
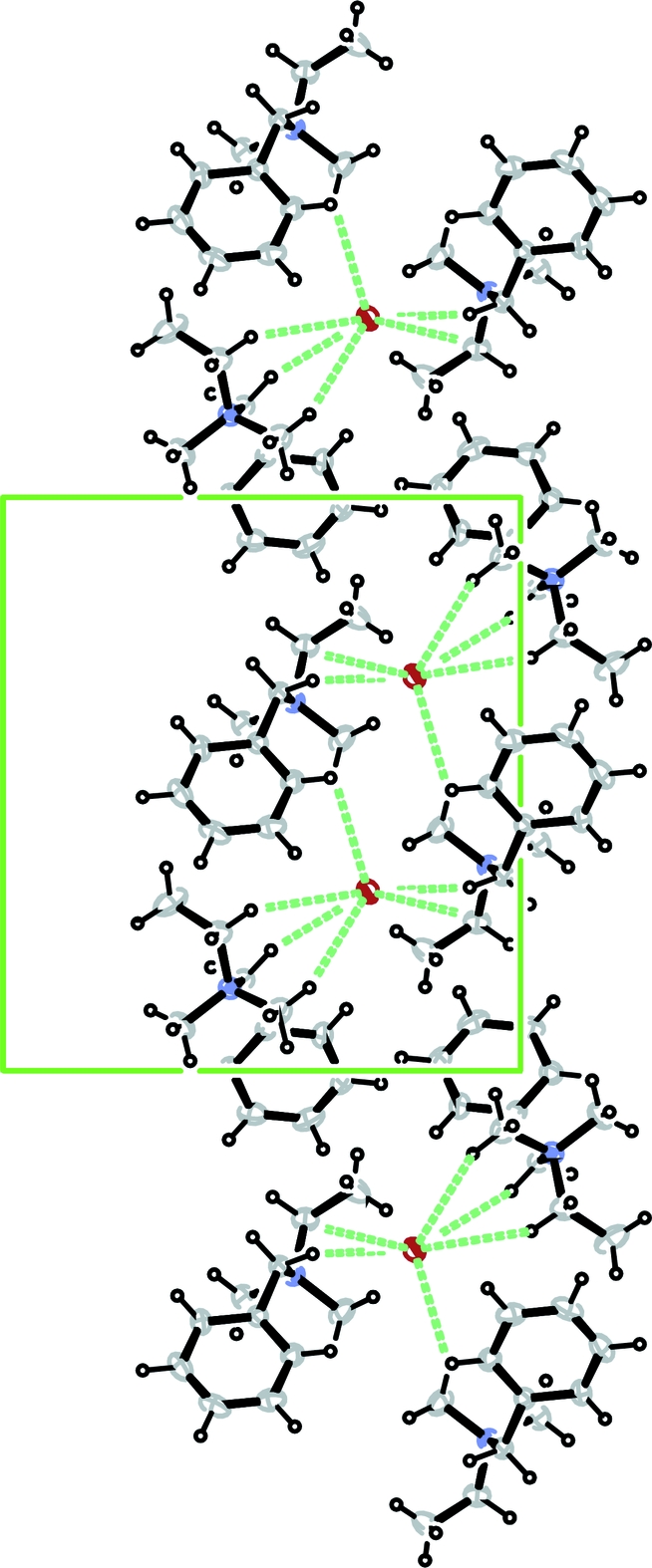
Layers parallel to (010) and built of the ammonium cations, arranged in a zig—zag 'head-to-tail' system, are joined together through Br counterions. View along [100] (ORTEP-3; Farrugia, 1997).

**Fig. 4.**
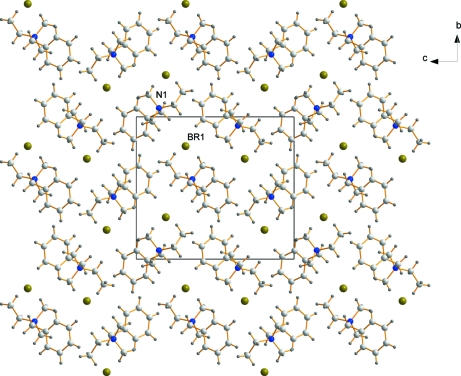
The sequence of the cationic and anionic layers along [010] in projecton onto (100) (DIAMOND; Brandenburg, 2006).

## supplementary crystallographic information

### Comment

Quaternary alkylammonium salts are widely used to modify natural clay minerals into hydrophobic organo-clays which exhibit high capability to remove hydrophobic contaminants from aqueous solutions (Ogawa & Kuroda, 1997). From the systematic study of the relation between the crystal structures of chosen homologous benzyldimethylalkylammonium bromides and their cations' ability for sorption on clay minerals (Kwolek *et al.*, 2003; Hodorowicz *et al.*, 2003, 2005), it became obvious that the hydrophobic interactions are responsible for an alkyl-chain bilayer formation when the long-chain (n = 8–12) ammonium cations are adsorbed on montmorillonite (Hodorowicz *et al.*, 2005), whereas a different way of cation packing seems to dominate in the case of short-chain ammonium cations (Kwolek *et al.*, 2003). The crystal structure analysis of benzyldimethylethylammonium bromide was performed to find out the influence of molecular geometry, and the length of the alkyl chain in particular, on the packing properties of the ammonium cations. The structure of the title compound is shown in Fig. 1. The asymmetric unit is composed of a quaternary ammonium cation and a bromide counterion (N^+^···Br^-^ = 4.439 (2) Å). The bond lengths and angles indicate the typical tetrahedral arragement of the substituents at the N atom. The molecular dimensions are comparable with the values reported in the literature (Allen *et al.*, 1987). Methyl and methylene groups of the quaternary ammonium cation as well as C—H of the benzene ring are involved in weak intermolecular interactions of the C—H···Br^-^ type (Table 1). There are also relatively strong interactions of the C—H···π type observed between the C2 methyl group and the π system of the benzene ring, which result in cation chains along [100] (Fig. 2). The chains are joined into layers parallel to (010) due to C—H···Br^-^ interactions (Fig. 3). The interactions are also responsible for packing of the layers along [010], as shown in Fig. 4. Each layer consists of cations inclined to the anionic layer and arranged in a zig—zag 'head-to-tail' system. The thickness of the layer is *b*/2. The observed architecture of the short-chain ammonium cation layers, best seen in Figs. 3 and 4, could be considered as a model for the organic cation layers intercalated into the montmorillonite structure (Kwolek *et al.*, 2003).

### Experimental

The title compound was prepared by dissolving a 1:1 mixture of bromoethane and *N*,*N*-dimethylbenzylamine in acetone at 273 K. The solution was slowly heated to room temperature to give colourless single crystals of the title compound. Recrystallization from acetone afforded crystals suitable for X-ray measurements.

### Refinement

All hydrogen atom positions were observed in a difference Fourier map. Nevertheless, in the refinement procedure the hydrogen atoms were positioned geometrically and refined using a riding model, with C—H = C—H = 0.97 Å for CH_2_ groups, 0.96 Å for CH_3_ groups, and 0.93 Å for aromatic CH, and with *U*_iso_(H) = 1.5*U*_eq_(C) for methyl groups and *U*_iso_(H) = 1.2*U*_eq_(C) for all other H atoms.

### Crystal data

**Table:**

C_11_H_18_N^+^·Br^–^	*F*_000_ = 504
*M**_r_* = 244.17	*D*_x_ = 1.364 Mg m^−^^3^
Orthorhombic, *P*2_1_2_1_2_1_	Mo *K*α radiation λ = 0.71073 Å
Hall symbol: P 2ac 2ab	Cell parameters from 2258 reflections
*a* = 6.7765 (1) Å	θ = 1.0–31.5º
*b* = 12.5827 (2) Å	µ = 3.42 mm^−^^1^
*c* = 13.9433 (2) Å	*T* = 293 (2) K
*V* = 1188.90 (3) Å^3^	Prism, colourless
*Z* = 4	0.20 × 0.19 × 0.17 mm

### Data collection

**Table:**

Nonius KappaCCD diffractometer	3874 independent reflections
Radiation source: fine-focus sealed tube	3483 reflections with *I* > 2σ(*I*)
Monochromator: horizontally mounted graphite crystal	*R*_int_ = 0.041
Detector resolution: 9 pixels mm^-1^	θ_max_ = 31.5º
*T* = 293(2) K	θ_min_ = 2.9º
φ and ω scans	*h* = 0→9
Absorption correction: multi-scan(DENZO and SCALEPACK Otwinowski & Minor, 1997)	*k* = 0→18
*T*_min_ = 0.548, *T*_max_ = 0.594	*l* = −20→20
18366 measured reflections	

### Refinement

**Table:**

Refinement on *F*^2^	H-atom parameters constrained
Least-squares matrix: full	*w* = 1/[σ^2^(*F*_o_^2^) + (0.0247*P*)^2^ + 0.2756*P*] where *P* = (*F*_o_^2^ + 2*F*_c_^2^)/3
*R*[*F*^2^ > 2σ(*F*^2^)] = 0.027	(Δ/σ)_max_ = 0.001
*wR*(*F*^2^) = 0.065	Δρ_max_ = 0.26 e Å^−^^3^
*S* = 1.07	Δρ_min_ = −0.49 e Å^−^^3^
3874 reflections	Extinction correction: SHELXL97 (Sheldrick, 2008), Fc^*^=kFc[1+0.001xFc^2^λ^3^/sin(2θ)]^-1/4^
119 parameters	Extinction coefficient: 0.045 (2)
Primary atom site location: structure-invariant direct methods	Absolute structure: Flack (1983), 1627 Friedel pairs
Secondary atom site location: difference Fourier map	Flack parameter: 0.002 (9)
Hydrogen site location: inferred from neighbouring sites	

### Special details

**Table:**

Geometry. All e.s.d.'s (except the e.s.d. in the dihedral angle between two l.s. planes) are estimated using the full covariance matrix. The cell e.s.d.'s are taken into account individually in the estimation of e.s.d.'s in distances, angles and torsion angles; correlations between e.s.d.'s in cell parameters are only used when they are defined by crystal symmetry. An approximate (isotropic) treatment of cell e.s.d.'s is used for estimating e.s.d.'s involving l.s. planes.
Refinement. Refinement of *F*^2^ against ALL reflections. The weighted *R*-factor *wR* and goodness of fit *S* are based on *F*^2^, conventional *R*-factors *R* are based on *F*, with *F* set to zero for negative *F*^2^. The threshold expression of *F*^2^ > σ(*F*^2^) is used only for calculating *R*-factors(gt) *etc*. and is not relevant to the choice of reflections for refinement. *R*-factors based on *F*^2^ are statistically about twice as large as those based on *F*, and *R*- factors based on ALL data will be even larger.

### Fractional atomic coordinates and isotropic or equivalent isotropic displacement parameters (Å^2^)

**Table:**

	*x*	*y*	*z*	*U*_iso_*/*U*_eq_	
Br1	0.29436 (3)	1.294368 (16)	0.812541 (14)	0.05290 (8)	
N1	0.7787 (2)	1.06178 (12)	0.85616 (10)	0.0397 (3)	
C1	0.8014 (4)	1.15508 (19)	0.92125 (17)	0.0613 (5)	
H1A	0.7328	1.2150	0.8947	0.074*	
H1B	0.9389	1.1720	0.9279	0.074*	
H1C	0.7471	1.1383	0.9830	0.074*	
C2	0.8857 (3)	0.9680 (2)	0.89734 (18)	0.0578 (5)	
H2A	0.8706	0.9081	0.8554	0.069*	
H2B	0.8317	0.9512	0.9591	0.069*	
H2C	1.0232	0.9848	0.9039	0.069*	
C31	0.7883 (5)	1.1763 (3)	0.70577 (19)	0.0808 (8)	
H31A	0.8569	1.1845	0.6460	0.097*	
H31B	0.8025	1.2399	0.7432	0.097*	
H31C	0.6509	1.1635	0.6936	0.097*	
C3	0.8734 (3)	1.08411 (19)	0.75980 (15)	0.0526 (5)	
H3A	1.0130	1.0972	0.7698	0.063*	
H3B	0.8617	1.0210	0.7203	0.063*	
C4	0.5616 (2)	1.03466 (14)	0.84108 (12)	0.0373 (3)	
H4A	0.4933	1.0982	0.8197	0.045*	
H4B	0.5518	0.9824	0.7902	0.045*	
C41	0.4578 (2)	0.99204 (13)	0.92838 (12)	0.0368 (3)	
C42	0.3755 (3)	1.05941 (16)	0.99624 (14)	0.0483 (4)	
H42	0.3879	1.1326	0.9892	0.058*	
C43	0.2748 (4)	1.0181 (2)	1.07459 (15)	0.0617 (5)	
H43	0.2222	1.0636	1.1205	0.074*	
C44	0.2528 (3)	0.9094 (2)	1.08438 (16)	0.0645 (6)	
H44	0.1860	0.8817	1.1370	0.077*	
C45	0.3293 (4)	0.84284 (19)	1.01657 (18)	0.0619 (6)	
H45	0.3126	0.7698	1.0229	0.074*	
C46	0.4319 (3)	0.88285 (15)	0.93829 (16)	0.0477 (4)	
H46	0.4832	0.8367	0.8925	0.057*	

### Atomic displacement parameters (Å^2^)

**Table:**

	*U*^11^	*U*^22^	*U*^33^	*U*^12^	*U*^13^	*U*^23^
Br1	0.06094 (12)	0.04546 (10)	0.05230 (11)	0.00357 (9)	0.00416 (9)	0.00747 (8)
N1	0.0369 (6)	0.0416 (7)	0.0405 (6)	−0.0039 (6)	−0.0001 (6)	−0.0024 (5)
C1	0.0611 (12)	0.0599 (12)	0.0629 (12)	−0.0199 (11)	0.0056 (12)	−0.0207 (10)
C2	0.0422 (9)	0.0646 (13)	0.0667 (13)	0.0038 (9)	−0.0115 (9)	0.0112 (10)
C31	0.0638 (13)	0.106 (2)	0.0727 (15)	0.0084 (16)	0.0168 (13)	0.0409 (14)
C3	0.0452 (9)	0.0642 (12)	0.0482 (10)	−0.0012 (9)	0.0100 (8)	0.0015 (9)
C4	0.0357 (7)	0.0384 (8)	0.0378 (7)	−0.0008 (6)	−0.0014 (6)	0.0000 (6)
C41	0.0356 (7)	0.0363 (8)	0.0386 (7)	−0.0027 (6)	−0.0026 (6)	0.0018 (6)
C42	0.0509 (9)	0.0470 (10)	0.0470 (9)	−0.0018 (8)	0.0061 (8)	−0.0034 (8)
C43	0.0592 (11)	0.0815 (15)	0.0444 (9)	−0.0056 (12)	0.0091 (10)	−0.0051 (10)
C44	0.0544 (13)	0.0901 (17)	0.0489 (10)	−0.0140 (11)	−0.0012 (8)	0.0251 (11)
C45	0.0603 (13)	0.0531 (11)	0.0722 (14)	−0.0119 (10)	−0.0057 (11)	0.0228 (10)
C46	0.0484 (9)	0.0363 (8)	0.0584 (10)	−0.0033 (7)	−0.0020 (9)	0.0034 (8)

### Geometric parameters (Å, °)

**Table:**

Br1—N1	4.4393 (16)	C3—H3B	0.970
N1—C1	1.492 (2)	C4—C41	1.505 (2)
N1—C2	1.499 (3)	C4—H4A	0.970
N1—C3	1.515 (2)	C4—H4B	0.970
N1—C4	1.525 (2)	C41—C42	1.387 (3)
C1—H1A	0.960	C41—C46	1.392 (2)
C1—H1B	0.960	C42—C43	1.389 (3)
C1—H1C	0.960	C42—H42	0.930
C2—H2A	0.960	C43—C44	1.382 (4)
C2—H2B	0.960	C43—H43	0.930
C2—H2C	0.960	C44—C45	1.365 (4)
C31—C3	1.498 (3)	C44—H44	0.930
C31—H31A	0.960	C45—C46	1.388 (3)
C31—H31B	0.960	C45—H45	0.930
C31—H31C	0.960	C46—H46	0.930
C3—H3A	0.970		
			
C1—N1—C2	109.66 (17)	C31—C3—H3A	108.5
C1—N1—C3	110.47 (16)	N1—C3—H3A	108.5
C2—N1—C3	106.30 (16)	C31—C3—H3B	108.5
C1—N1—C4	111.07 (16)	N1—C3—H3B	108.5
C2—N1—C4	110.08 (15)	H3A—C3—H3B	107.5
C3—N1—C4	109.15 (14)	C41—C4—N1	114.79 (13)
C1—N1—Br1	68.95 (13)	C41—C4—H4A	108.6
C2—N1—Br1	158.26 (12)	N1—C4—H4A	108.6
C3—N1—Br1	93.99 (11)	C41—C4—H4B	108.6
C4—N1—Br1	54.22 (8)	N1—C4—H4B	108.6
N1—C1—H1A	109.5	H4A—C4—H4B	107.5
N1—C1—H1B	109.5	C42—C41—C46	119.00 (18)
H1A—C1—H1B	109.5	C42—C41—C4	121.45 (15)
N1—C1—H1C	109.5	C46—C41—C4	119.40 (16)
H1A—C1—H1C	109.5	C41—C42—C43	120.3 (2)
H1B—C1—H1C	109.5	C41—C42—H42	119.8
N1—C2—H2A	109.5	C43—C42—H42	119.8
N1—C2—H2B	109.5	C44—C43—C42	120.1 (2)
H2A—C2—H2B	109.5	C44—C43—H43	120.0
N1—C2—H2C	109.5	C42—C43—H43	120.0
H2A—C2—H2C	109.5	C45—C44—C43	119.8 (2)
H2B—C2—H2C	109.5	C45—C44—H44	120.1
C3—C31—H31A	109.5	C43—C44—H44	120.1
C3—C31—H31B	109.5	C44—C45—C46	120.8 (2)
H31A—C31—H31B	109.5	C44—C45—H45	119.6
C3—C31—H31C	109.5	C46—C45—H45	119.6
H31A—C31—H31C	109.5	C45—C46—C41	119.9 (2)
H31B—C31—H31C	109.5	C45—C46—H46	120.0
C31—C3—N1	115.22 (18)	C41—C46—H46	120.0
			
C1—N1—C3—C31	−60.9 (3)	N1—C4—C41—C46	−98.94 (19)
C2—N1—C3—C31	−179.8 (2)	C46—C41—C42—C43	2.2 (3)
C4—N1—C3—C31	61.5 (2)	C4—C41—C42—C43	177.88 (19)
Br1—N1—C3—C31	8.1 (2)	C41—C42—C43—C44	−1.2 (3)
C1—N1—C4—C41	−68.2 (2)	C42—C43—C44—C45	−0.4 (4)
C2—N1—C4—C41	53.4 (2)	C43—C44—C45—C46	0.9 (4)
C3—N1—C4—C41	169.73 (15)	C44—C45—C46—C41	0.1 (3)
Br1—N1—C4—C41	−109.45 (15)	C42—C41—C46—C45	−1.6 (3)
N1—C4—C41—C42	85.4 (2)	C4—C41—C46—C45	−177.40 (18)

### Hydrogen-bond geometry (Å, °)

**Table:**

*D*—H···*A*	*D*—H	H···*A*	*D*···*A*	*D*—H···*A*
C1—H1A···Br1	0.96	3.34	4.144 (3)	143
C4—H4A···Br1	0.97	2.81	3.757 (2)	164
C42—H42···Br1	0.93	3.26	3.950 (2)	132
C31—H31C···Br1	0.96	3.36	3.953 (3)	122
C2—H2A···Br1^i^	0.96	2.96	3.850 (3)	154
C4—H4B···Br1^i^	0.97	2.96	3.832 (2)	151
C3—H3B···Br1^i^	0.97	3.08	3.950 (2)	151
C3—H3A···Br1^ii^	0.97	3.19	3.959 (2)	138
C1—H1C···Br1^iii^	0.96	2.99	3.766 (2)	139
C2—H2C···Cg1^ii^	0.96	2.69	3.526	145

Symmetry codes: (i) −*x*+1, *y*−1/2, −*z*+3/2; (ii) *x*+1, *y*, *z*; (iii) *x*+1/2, −*y*+5/2, −*z*+2.
